# GABA_A_ Receptors Are Well Preserved in the Hippocampus of Aged Mice

**DOI:** 10.1523/ENEURO.0496-18.2019

**Published:** 2019-08-14

**Authors:** Thulani H. Palpagama, Mélanie Sagniez, SooHyun Kim, Henry J. Waldvogel, Richard L. Faull, Andrea Kwakowsky

**Affiliations:** Centre for Brain Research, Department of Anatomy and Medical Imaging, Faculty of Medical and Health Sciences, University of Auckland, Auckland 1142, New Zealand

**Keywords:** ageing, GABA_A_ receptor, hippocampus, mouse

## Abstract

GABA is the primary inhibitory neurotransmitter in the nervous system. GABA_A_ receptors (GABA_A_Rs) are pentameric ionotropic channels. Subunit composition of the receptors is associated with the affinity of GABA binding and its downstream inhibitory actions. Fluctuations in subunit expression levels with increasing age have been demonstrated in animal and human studies. However, our knowledge regarding the age-related hippocampal GABA_A_R expression changes is limited and based on rat studies. This study is the first analysis of the aging-related changes of the GABA_A_R subunit expression in the CA1, CA2/3, and dentate gyrus regions of the mouse hippocampus. Using Western blotting and immunohistochemistry we found that the GABAergic system is robust, with no significant age-related differences in GABA_A_R α1, α2, α3, α5, β3, and γ2 subunit expression level differences found between the young (6 months) and old (21 months) age groups in any of the hippocampal regions examined. However, we detected a localized decrease of α2 subunit expression around the soma, proximal dendrites, and in the axon initial segment of pyramidal cells in the CA1 and CA3 regions that is accompanied by a pronounced upregulation of the α2 subunit immunoreactivity in the neuropil of aged mice. In summary, GABA_A_Rs are well preserved in the mouse hippocampus during normal aging although GABA_A_Rs in the hippocampus are severely affected in age-related neurological disorders, including Alzheimer’s disease.

## Significance Statement

The current knowledge on GABAergic age-related alterations across different regions of the mouse brain is limited. These findings highlight that hippocampal GABA_A_R subunit composition and receptor function is well preserved in the hippocampus during normal aging in mice. Aging is the main risk factor for Alzheimer’s disease and other neurodegenerative disorders characterized by established remodeling of the hippocampal GABAergic system. Mice are frequently used as disease models of aging, and the majority of the transgenic animal-based research on neurodegenerative conditions has been conducted with mouse models, but the age-related GABA_A_R subunit expression changes have not been examined in the mouse brain. Therefore, studies like this are necessary to understand the importance of age in study design and interpretation of results.

## Introduction

GABA is the primary inhibitory neurotransmitter in the nervous system and the dysregulation of GABA signaling in aging is well established (Lehmann et al., 2012; McQuail et al., 2015; Rozycka and Liguz-Lecznar, 2017). Age-related alterations affect specific neuronal subpopulations and their synaptic contacts but the direction of these changes are variable in different brain areas. Whereas the prefrontal cortex exhibits increased inhibition with age, data suggest decreased intracortical inhibition in sensory systems and the hippocampus (Luebke et al., 2004; Potier et al., 2006; Schmidt et al., 2010; Lehmann et al., 2012; Cheng and Lin, 2013; Bañuelos et al., 2014; McQuail et al., 2015). The fine balance between excitatory and inhibitory circuits is fundamental for neuroplasticity and all aspects of brain function. Age-specific GABA signaling alterations might disturb this balance and change vulnerability to disease conditions such as, depression, anxiety, presbycusis, epilepsy, and Alzheimer`s disease (Mohler, 2006; Sharashenidze et al., 2007; Rissman and Mobley, 2011; McQuail et al., 2015; Flores-Ramos et al., 2017; Fuhrer et al., 2017; Govindpani et al., 2017; Rozycka and Liguz-Lecznar, 2017; Kwakowsky et al., 2018a,b).

GABA is synthesized by glutamic acid decarboxylase (GAD) and is then recruited into synaptic vesicles. Following membrane depolarization, GABA is released into the synapse and binds to either ionotropic GABA_A_ receptors (GABA_A_Rs) or metabotropic GABA_B_ receptors (GABA_B_Rs). GABA_A_Rs are ligand dependent Cl^−^ channel pores assembled from five subunits (Sigel and Steinmann, 2012). Over 20 GABA_A_R subunits have been identified; six alpha subunits (α1/2/3/4/5/6), three beta subunits (β1/2/3), three gamma subunits (γ1/2/3), delta (δ), theta (θ), epsilon (ε), pi (π), and rho (ρ1/2/3), forming many possible combinations of pentameric GABA_A_Rs (Sieghart et al., 1999; Chen and Olsen, 2007; Sieghart and Savić, 2018). The expression pattern of subunits is brain region specific and is involved in region-specific function (McKernan and Whiting, 1996; Olsen and Sieghart, 2009). Previous studies have reported aging-related alterations of GABA, GAD, and GABAR levels in different species and brain areas (Milbrandt et al., 1994; Turgeon and Albin, 1994; Caspary et al., 1995, 2013; Loerch et al., 2008; Rissman and Mobley, 2011; Long et al., 2013; Bañuelos et al., 2014; Liguz-Lecznar et al., 2015; McQuail et al., 2015; He et al., 2016; Porges et al., 2017; Rozycka and Liguz-Lecznar, 2017; Pandya et al., 2018). There is also growing evidence to suggest regional brain function loss, hearing impairment, and learning and memory deficits, as an implication of regional GABA_A_R subunit expression changes in aging (Caspary et al., 1995, 2013; Rissman and Mobley, 2011; Govindpani et al., 2017). However, most results supporting these findings come from rat studies. Despite the fact that mice are frequently used as disease models of aging, and that the majority of the transgenic animal-based research on neurodegenerative conditions has been conducted with mouse models, the age-related GABA_A_R subunit expression changes have not been reported in the mouse brain.

Aging is the main risk factor for Alzheimer’s disease and other neurodegenerative disorders and have also been linked to decline in the GABAergic system (Rissman and Mobley, 2011; Fuhrer et al., 2017; Govindpani et al., 2017). Therefore, a thorough investigation is required to identify the link between age and the GABAergic changes observed in these neurologic conditions, for better understanding of disease prevalence, progression, and finding new treatment strategies. The hippocampus is severely affected in Alzheimer’s disease and shows many age-related molecular and cellular changes (West et al., 1994; Serrano-Pozo et al., 2011; Moodley and Chan, 2014). Accordingly, for appropriate study design and interpretation of findings from mouse studies it is critical to examine the vulnerability of the GABA_A_R subunits to aging in the hippocampus.

This study is the first analysis of the age-specific changes of the GABA_A_R subunit expression in the mouse hippocampus. In the present study we did not observe any significant alterations in the expression of GABA_A_R α1, α2, α3, α5, β3, and γ2 subunits in the mouse CA1, CA2/3, and dentate gyrus (DG) hippocampal regions using quantitative Western blotting and immunohistochemistry. However, the α2 subunit displayed decreased expression around the soma, proximal dendrites, and in the axon initial segment of pyramidal cells in the CA1 and CA3 regions and upregulation in the neuropil of aged mice. These findings suggest that GABA_A_R subunit expression in the mouse hippocampus is well protected against age-related alterations.

## Methods

### Animals and brain tissue preparation

All experiments were approved and performed in accordance with the regulations of the University of Otago and University of Auckland. All mice were bred and housed at the Hercus Taieri Resource Unit, University of Otago and Vernon Jansen Unit, University of Auckland. The animals were maintained under conditions of a 12 h light/dark cycle (lights on at 7:00 A.M.) with food and water available *ad libitum*. All experiments were performed on young (6 months; *n* = 6) and old (21 months; *n* = 6) C57BL/6 wild-type male mice.

Processing of tissue for Western blotting followed the described procedure (Spijker, 2011). First, the brain was cut in half separating the hemispheres on ice; the hippocampus was dissected from each hemisphere of the brain and microdissected into the CA1, CA2/3, and DG hippocampal regions, and then freshly snap-frozen on dry ice and stored at −80° C.

For immunohistochemistry experiments mice were deeply anesthetized with an overdose of 75 mg/kg ketamine and 1 mg/kg domitor (Pfizer) and perfused transcardially with 20 ml of ice-cold 4% paraformaldehyde in phosphate buffer, pH 7.6. Brains were removed and postfixed in paraformaldehyde solution for 2 h at room temperature (RT) and then incubated in 30% sucrose in Tris-phosphate saline (TBS; pH 7.6, 0.05 m Tris, 0.15 m NaCl) solution overnight at 4°C. Four sets of 30-μm-thick coronal brain sections were cut using a freezing microtome.

### Western blotting

The fresh mouse hippocampal tissue samples were collected from the regions-of-interest, homogenized in a buffer containing 0.5 m Tris, 100 mm EDTA, 4% SDS, pH 6.8, supplemented with a protease inhibitor cocktail (P8340, Sigma-Aldrich) and 100 mm phenylmethanesulfonyl fluoride (P7626, Sigma-Aldrich), and protein extracts prepared using 0.5 mm glass beads (Mo Bio) and a Mini Bullet Blender Tissue Homogenizer (Next Advance) at speed 8 for 8 min. The homogenates were incubated for 1 h on ice, and then centrifuged at 10,000 rpm for 10 min and the supernatant collected and stored at −20°C. The protein concentration of the samples was measured using detergent-compatible protein assay (DC Protein assay, 500-0116, Bio-Rad), following the manufacturer’s instructions. Protein samples from each mouse were randomized, by a person not involved in the study, and numbered from 1 to 12. Twenty to forty μg of each protein extract was run on a gradient SDS PAGE gel (NU PAGE 4–12% BT 1.5, NP0336BOX, Life Technologies) and then blotted. Proteins were separated in XCell SureLock Mini-Cell system (Invitrogen) and transferred onto nitrocellulose membranes using XCell Blot Module (Invitrogen). Two molecular weight ladders, Precision and SeeBlue (Life Technologies), were also loaded in gels as verification of labeled band size. Membranes were blocked with Odyssey blocking buffer (LI-COR Biosciences) at RT for 30 min, followed by incubation with the primary antibodies (Table 1) at 4°C overnight. The following day membranes were washed 3× 10 min in Tris-buffered saline pH 7.6, 0.1% Tween, and incubated with an appropriate IRDye (1:10,000; goat anti-rabbit IRDye 680RD, 926-68071; RRID:AB_10956166; goat anti-mouse IRDye 800CW, 926-32210; RRID:AB_621842; donkey anti-goat IRDye 800CW, 926-32214; RRID:AB_621846; LI-COR Biosciences) secondary antibody for 1 h at RT. Membranes were washed and scanned on an Odyssey Infrared Imaging System (LI-COR Biosciences). All antibody dilutions were optimized.

**Table 1.** Primary antibodies used in this study

**Antigen**	**Host**	**Source, catalog #**	**Concentration WB**	**Concentration IHC**
α_1_	Rabbit	Alomone Labs, AGA-001	1:1000	1:1000
α_2_	Rabbit	Alomone Labs, AGA-002	1:200	1:100
α_3_	Rabbit	Alomone Labs, AGA-003	1:200	1:200
α_5_	Rabbit	ThermoFisher Scientific, PA5-31163	1:200	1:200
β_3_	Mouse	Novus, NBP-1-47613	1:1000	1:500
γ_2_	Goat	Santa Cruz Biotechnology, SC-131935	1:250	1:250
Beta actin	Rabbit	Abcam, ab8227	1:1000	
Beta actin	Mouse	Abcam, ab6276	1:1000	

WB, Western blot; IHC, immunohistochemistry.

The immunofluorescence signal was detected at 680 and 800 nm spectrum using the Odyssey Infrared Imaging System (LI-COR Biosciences). The analyses were conducted using the Image Studio Lite software v5.2 (LI-COR Biosciences) to measure signal intensities of each sample and were normalized to β-actin.

### Immunohistochemistry

Free-floating single-label fluorescence immunohistochemistry was performed to detect GABA_A_R α1, α2, α3, α5, β3, and γ2 subunits within the mouse hippocampus. Brain sections were first incubated in blocking solution containing TBS, 0.25% bovine serum albumin, 0.3% Triton X-100, and 1% donkey serum for 1 h at RT followed by incubation with one of the primary antibodies (Table 1) for 48 h at 4°C. The sections were then incubated in either biotinylated donkey anti-rabbit, anti-mouse, or anti-goat IgG (1:200; Sigma-Aldrich) for 2 h at RT followed by streptavidin AlexaFluor 647 (1:500; ThermoFisher Scientific) incubation for 1 h at RT. Nuclei were counterstained with Hoechst 33342 (1:10,000; Invitrogen). Sections were then mounted on slides, air dried overnight, and coverslipped with Mowiol mounting medium. Omission of primary antibodies resulted in a complete absence of immunoreactivity.

Imaging was conducted using a Zeiss 710 confocal laser-scanning microscope (Carl Zeiss). The experimenter was blinded to avoid any potential bias during image acquisition and analysis. Integrated density measurements were undertaken using ImageJ, with the size of the measured areas as follows: 21,352μm^2^ for the CA1 region, 4761μm^2^ for the CA3 region, and 12,295μm^2^ for the DG in each layer. Intensity measurements were taken in the regions of the stratum (str.) pyramidale, str. radiatum, and str. moleculare of the CA1 and CA3 regions, and the hilus, str. moleculare, and str. granulosum of the DG.

### Statistical analysis

Data in all experiments were expressed as mean ± SEM. To examine the averaged signal intensity and integrated density differences between groups [young (*n* = 6) vs old (*n* = 6) males] an unpaired Mann–Whitney test was used. All statistical analyses were conducted using Prismv6 (GraphPad Software; RRID:SCR_002798) with a value of *p* < 0.05 considered significant. Photoshop CC 2017 (Adobe) was used to prepare the figures.

## Results

**Figure 1.**
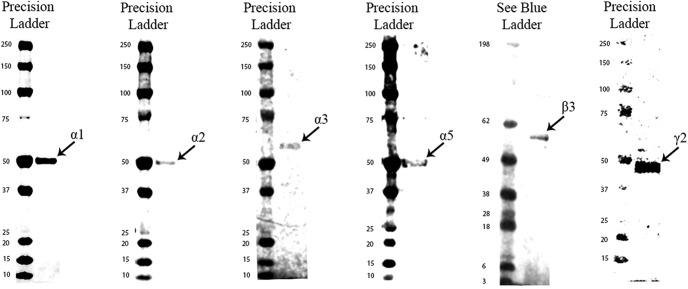
Western blot against mouse hippocampal protein homogenates probed with GABA_A_R α1, α2, α3, α5, β3, and γ2 subunit antibodies. Each lane has 20–40 μg of protein loaded. Observed band sizes: α1, α2, α5: ∼52 kDa; α3, β3: ∼53 kDa; γ2: ∼44 kDa.

**Figure 2.**
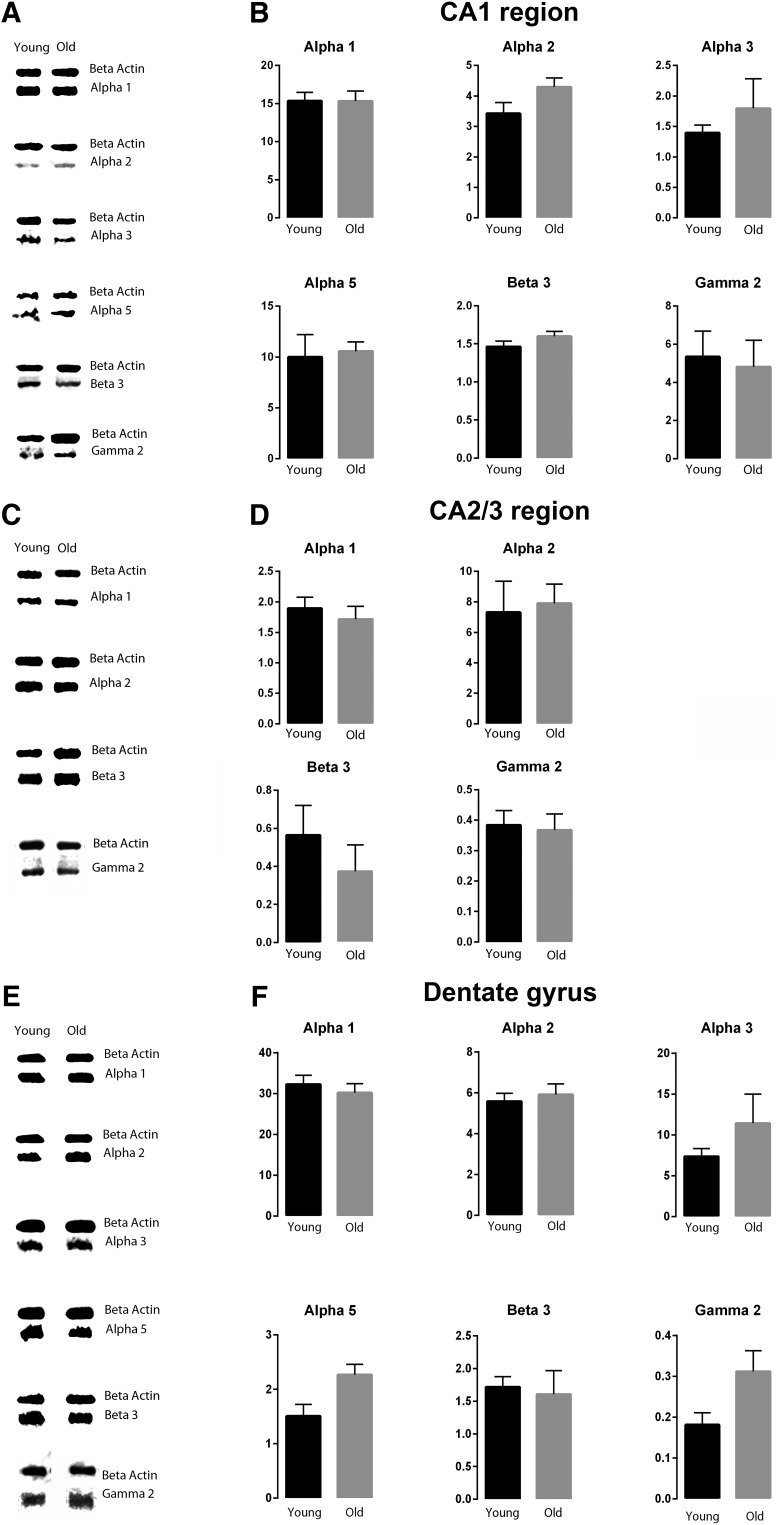
Representative immunoreactive Western blot bands from young male (Young) and old male (Old) hippocampal CA1, CA2/3, and DG homogenates following incubation with antibodies to the GABA_A_R α1, α 2, α 3, α 5, β3, and γ2 subunits (***A***, CA1; ***C***, CA2/3; ***E***, DG) and corresponding signal intensity graphs (***B***, CA1; ***D***, CA2/3; ***F***, DG). Signal intensity for each GABA_A_R subunit Western blot band was measured and normalized to their corresponding β-actin signal for each age group. The data are graphed as mean ± SEM (*n* = 6, with 3 replicates; unpaired Mann–Whitney test).

**Figure 3.**
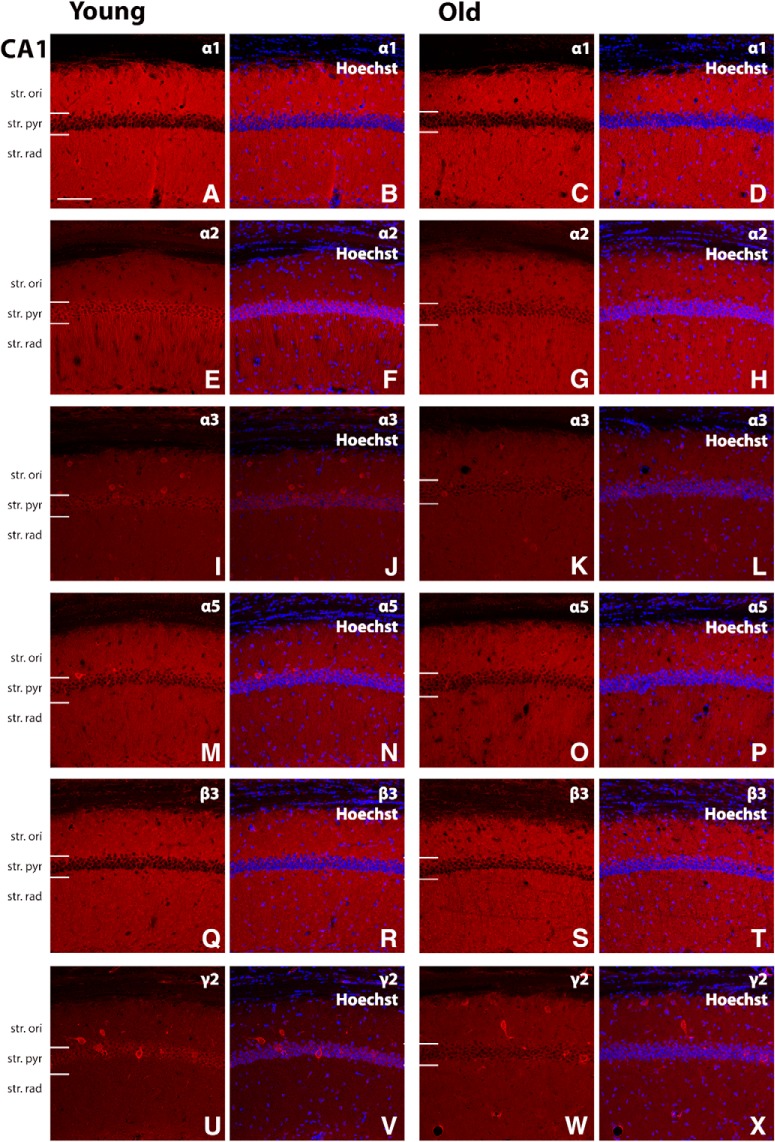
Representative photomicrographs of the CA1 region showing GABA_A_R α1, α2, α3, α5, β3, and γ2 subunit expression (red) and α1, α2, α3, α5, β3, and γ2 immunoreactivity overlaid with Hoechst (blue) labeling for representative young and old mice (***A–X***). The strong α2 subunit immunoreactivity is evident around the soma, proximal dendrites, and possibly in the axon-initial segment of individual pyramidal cells of the CA1 region in young animals but the labeling is decreased at these sites in aged mice (***E–H***). Scale bar, 50 μm. Startum (str), oriens (ori), pyramidale (pyr), radiatum (rad).

**Figure 4.**
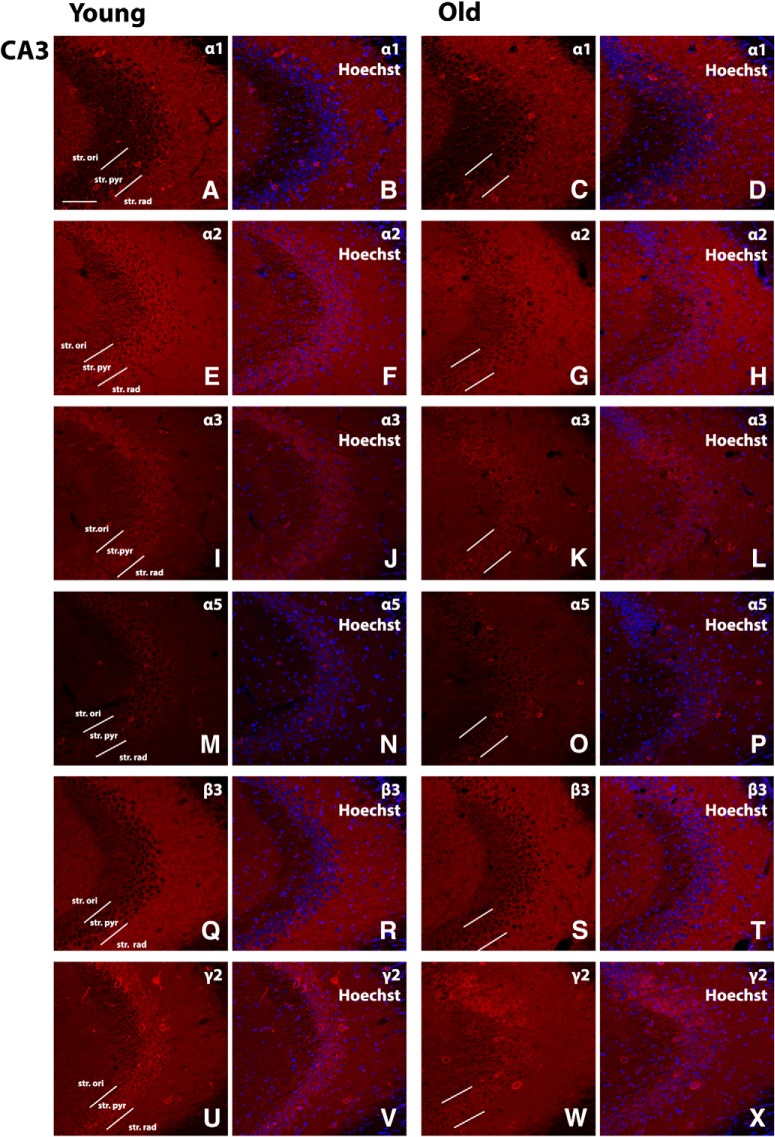
Representative photomicrographs of the CA3 region showing GABA_A_R α1, α2, α3, α5, β3, and γ2 subunit expression (red), and α1, α2, α3, α5, β3, and γ2 immunoreactivity overlaid with Hoechst (blue) labeling for representative young and old mice (***A–X***). Scale bar, 50 μm.

**Figure 5.**
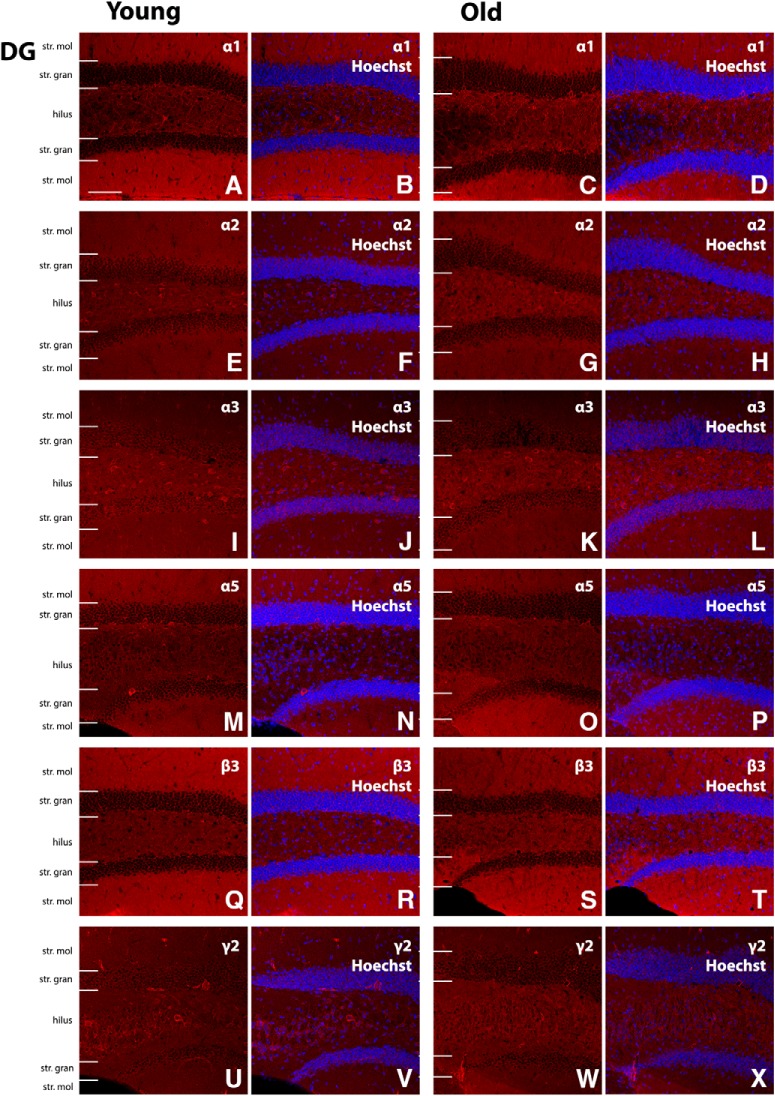
Representative photomicrographs of the DG region showing GABA_A_R α1, α2, α3, α5, β3, and γ2 subunit expression (red), and α1, α2, α3, α5, β3, and γ2 immunoreactivity overlaid with Hoechst (blue) labeling for representative young and old mice (***A–X***). Scale bar, 50 μm. Moleculare (mol), granulare (gran).

**Figure 6.**
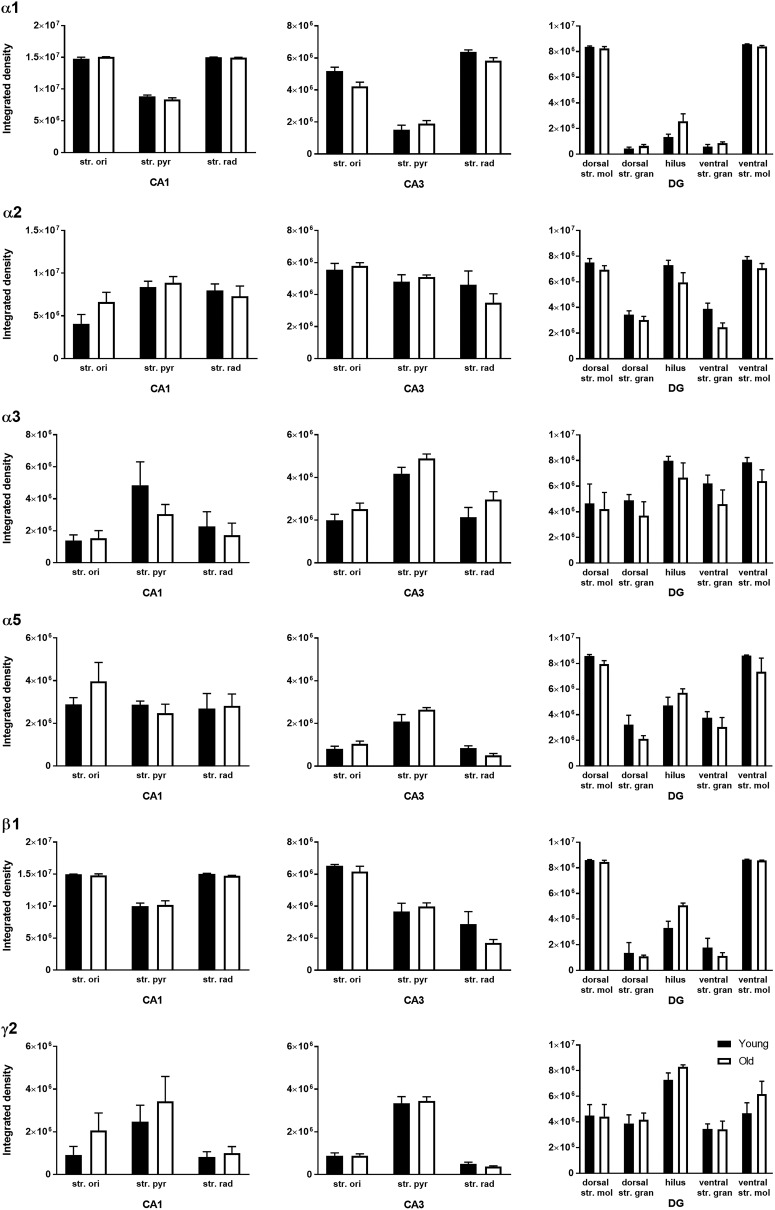
Quantification of GABA_A_R α1, α2, α3, α5, β3, and γ2 subunit immunoreactivity in the regions and layers of the hippocampus in young and old mice. The data are graphed as mean ± SEM (*n* = 6; unpaired Mann–Whitney test).

The expression levels of GABA signaling components, GABA_A_R α1, α2, α3, α5, β3, and γ2 subunits, were examined by Western blotting and immunohistochemistry in the mouse hippocampal CA1, CA2/3, and DG regions of young and old mice. For all antibodies used, the expected band sizes were detected (Fig. 1). The GABA_A_R α1 and β3 subunits displayed the highest expression level within all the hippocampal regions examined, while the α2 and α3 subunits were expressed at low levels within the CA1 region (Figs. 2*A*,*B*, 3*A*–*T*, 4*A–T*, 5*A–T*). The large clusters of α2 subunit immunoreactivity is evident around the soma, proximal dendrites and possibly in the axon initial segment of individual pyramidal cells of the CA1 and CA3 regions in young animals but the labeling is specifically decreased at these sites in aged mice (Figs. 3*E*–*H*, 4*E*–*H*). However, a pronounced upregulation was detected for the α2 subunit in the neuropil of the CA1 and CA3 regions (Figs. 3*E*–*H*, 4*E*–*H*). Because of this upregulation in the neuropil despite the downregulation around the soma, proximal dendrites and possibly in the axon initial segment there was no altered α2 subunit expression found between the young and aged samples (CA1 str ori: 4.1 × 10^6^ ± 108,142 vs 6.6 × 10^6^ ± 1141,847, *p* = 0.13; CA1 str pyr: 8.4 × 10^6^ ± 695,258 vs 6.3 × 10^6^ ± 753,969, *p* = 0.94; CA1 str rad: 7.98 × 10^6^ ± 764,725 vs 7.3 × 10^6^ ± 1199,600, *p* = 0.82; CA3 str ori: 5.5 × 10^6^ ± 396,001 vs 5.8 × 10^6^ ± 209,811, *p* = 0.84; CA3 str pyr: 4.8 × 10^6^ ± 437,527 vs 5.1 × 10^6^ ± 124,709, *p* > 0.99; CA3 str rad: 4.6 × 10^6^ ± 857,431 vs 3.5 × 10^6^ ± 562,171, *p* = 0.42; *n* = 6) when examined with densitometry analysis (Fig. 6). In the CA2/3 regions the staining intensity was below the detection limit for the α3 and α5 subunits in Western blotting experiments and showed weak immunolabeling (Fig. 4*I*–*P*).

In the mouse hippocampal CA1, CA2/3, and DG regions, the GABA_A_R α1, α2, α3, α5, β3, and γ2 subunits were well preserved during aging (Figs. 2–6). These hippocampal regions did not show significant changes in the expression level of any of the GABA_A_R subunits examined between the two age groups (Figs. 2–6).

## Discussion

In this study, we report that GABA_A_Rs are generally robustly preserved against age-related alterations in the mouse hippocampal CA1, CA2/3, and DG regions.

Our current knowledge on GABAergic age-related alterations across different regions of the mouse brain is limited and this is the first study to explore the age-specific expression of GABA_A_R subunits in the mouse hippocampus. Previous literature suggests that GABAergic changes may consequentially lead to compensatory changes for maintaining homeostasis of neuronal circuits and may affect the regional neuronal network functionality (Kralic et al., 2006; Fritschy, 2008; Rissman and Mobley, 2011; McQuail et al., 2015; Fuhrer et al., 2017; Rozycka and Liguz-Lecznar, 2017; Kwakowsky et al., 2018a,b). The differential expression pattern of GABA_A_R subunits affects GABA binding affinity, functioning of the receptor and alters the activity of downstream neuronal networks (Sieghart et al., 1999). Hence, it is critical to determine which brain regions are affected during the aging process.

We found no age-related changes in the expression level of α1, α2, α3, α5, β3, or γ2 subunits in the CA1 region of the mouse hippocampus. There is evidence in the literature to support the lack of age-related expression change of GABA_A_R subunits β3 and γ2 in the CA1 region of other species (Miralles et al., 1994; Gutiérrez et al., 1996; Rissman et al., 2006). Gutiérrez et al. (1996) showed no changes in GABA_A_R β2, β3, and γ2 subunits in the CA1 region of the aged rat hippocampus using *in situ* hybridization and immunocytochemistry. Immunolabeling of β2/3 in aged monkeys showed marked intersubject variability in labeling intensity, with dramatic reductions observed in 3 of 5 samples (Rissman et al., 2006). Interestingly, we observed a similar variability in β3 subunit expression in the mouse CA2/3 regions. The underlying mechanisms are unknown but environmental factors like stress, have been reported to influence GABA_A_R subunit expression changes in different brain areas, including the hippocampus (Skilbeck et al., 2010; Jie et al., 2018; Nejatbakhsh et al., 2018). No age-related expression data for subunits α2 and α3 have been previously published and the results are controversial regarding the age-related α1 subunit expression level changes. Yu et al. (2006) observed stable α1 subunit density during aging in the rat hippocampus using immunohistochemistry and densitometry, whereas a quantitative *in situ* hybridization study has demonstrated a significantly increased (34%) α1 subunit mRNA expression in the hippocampus of old rats (Gutiérrez et al., 1996). The largest increases were observed in the DG (76%) and in the CA1 region (30%), whereas the expression remained unchanged within the CA2 and CA3 regions (Gutiérrez et al., 1996). Another study also confirmed an increase in α1 subunit expression with aging in the rat hippocampus homogenate at mRNA and protein level (Ruano et al., 2000). However, these findings were contradicted by studies which found an age-dependent decrease in the α1 subunit expression in the CA1 region of the rhesus monkey and human hippocampus using immunohistochemistry combined with densitometric analysis (Kanaumi et al., 2006; Rissman et al., 2006).

In agreement with our findings, in the mouse and rat hippocampus α2 subunit expression has also been reported to show a relatively strong immunoreactivity in the pyramidal layer, particularly abundant in the axon initial segment, and strong diffuse staining in other layers of the CA1 and CA3 regions (Fritschy and Mohler, 1995; Nusser et al., 1996; Fritschy et al., 1998; Bouilleret et al., 2000). The decreased expression of α2 subunit around the soma, proximal dendrites, and in the axon initial segment of pyramidal cells in the CA1 and CA3 regions is not due to cell loss because the number of pyramidal neurons did not change as it has been revealed by the nuclear marker and the staining that was weaker but still clearly outlined the pyramidal cell bodies and dendritic processes. The pronounced upregulation of the α2 subunit immunoreactivity in the neuropil of the CA1 and CA3 regions explains why there is no altered α2 subunit expression between the young and aged samples when examined with Western blotting and densitometry based on immunohistochemistry.

In the cerebral cortex and hippocampus, the α2 subunits are highly expressed on pyramidal cells. The perisomatic α2-containing GABA_A_Rs mainly mediate the synaptic inhibitory input arising from CCK-positive basket cells and at the axon initial segment they mediate the GABAergic input from Chandelier cells, interneurons that control the firing pattern of principal cells by suppressing action potential propagation (Fritschy and Mohler, 1995; Nusser et al., 1996; Fritschy et al., 1998). The α2 subunit containing GABA_A_R subtype mediates anxiolytic-like, reward-enhancing, and antihyperalgesic actions of diazepam, and has antidepressant-like properties (Mohler, 2006; Engin et al., 2012; Smith et al., 2012). The functional significance of age-related altered α2 subunit immunoreactivity in the CA1 and CA3 regions is not known and requires further investigations. However, GABAergic tone will be very likely compromised in the aged hippocampus because of these age-related alterations in α2 subunit expression pattern, leading to altered anxiety/depression-related behaviors (Mohler, 2006; Engin et al., 2012), and learning and memory impairments (Buzsáki and Chrobak, 1995; Paulsen and Moser, 1998), a phenomenon that occurs during normal aging (Barnes, 1994; Vela et al., 2003). α2-containing GABA_A_Rs have also been implicated in schizophrenia-related cognitive impairments. The α2 subunit is upregulated in the axon initial segments in the dorsolateral prefrontal cortex of individuals with schizophrenia and major depressive disorder compared with matched control subjects (Lewis et al., 2005). The α2 subunit shows altered subregion and layer-specific expression in the Alzheimer’s disease hippocampus and temporal lobe and might contribute to network dysfunction and cognitive deficits during the progression of the disease (Limon et al., 2012; Kwakowsky et al., 2018a,b). The reduction of the α2 subunit has also been reported in brains of autistic patients, suggesting a possible linkage of this subunit in cognitive deficits unrelated to aging (Fatemi et al., 2009).

Cognitive processes involve neuronal networks in synchronous rhythmic activity that is controlled by inhibitory interneuron firing. Axon-initial segment and perisomatic synapses are important for synchronization of large populations of pyramidal neurons. Altered α2 subunit expression at the axon-initial segment and around the soma could have significant consequences for the efficacy and timing of GABAergic hyperpolarization, and previous studies suggest any alterations in the kinetics of GABAergic responses will alter the power and frequency of γ-oscillations (Gingrich et al., 1995; Ortinski et al., 2004; Traub et al., 2004; Hines et al., 2013). Therefore, α2-containing GABA_A_Rs might be important determinants of cortical and hippocampal network activity and working memory.

In the DG, our results showed an increasing trend, which did not reach statistical significance, of the γ2 subunit expression in older mice compared with the younger group. A similar trend was also observed in a study conducted by Ruano et al. (2000) when examining the expression of the short version of the γ2 subunit in the aged rat hippocampus, but this increase was not observed for the mature receptor. Conversely, another rat study reported a sustained downregulation of the γ2 subunit from P30 continuing during aging (Yu et al., 2006). Whereas the mouse CA1 and CA2/3 regions did not show γ2 subunit alteration with aging, in the CA1 region of the human hippocampus a moderate increase in the γ2 subunit level (12 vs 46–75 years old) was reported as revealed by immunohistochemistry and cell density analysis (Kanaumi et al., 2006). However, the limitation of this study is that differences between an adult and older age group were not examined and this makes it difficult to compare our results with these findings.

We also observed an age-related trend toward an increase in α5 subunit expression in the DG of older mice that did not reach statistical significance. Conversely, one rat study reported a moderate decrease in α5 subunit expression in the hippocampus during aging at protein level (Yu et al., 2006), but another study did not find any age-related changes at mRNA level (Ruano et al., 2000). The α5 subunit is highly expressed in the hippocampus and several studies using pharmacological agents and genetic manipulations have demonstrated that the α5 subunit plays a role in hippocampus-dependent learning (Collinson et al., 2002; Crestani et al., 2002; Chambers et al., 2004; Yee et al., 2004). An increased expression of the α5 subunit in the hippocampus is associated with memory loss (Wang et al., 2012), suggesting that upregulated expression of this subunit with aging might underlie age-related cognitive changes and vulnerability to age-related disease conditions (Potier et al., 1992; Barnes, 1994).

The discrepancy in results regarding age-related changes in hippocampal GABA_A_R subunit expression might be because of experimental design and species-specific changes. Most studies looked at the GABA_A_R subunit changes in the whole hippocampus, tissue homogenate, or density measured in the entire hippocampus, as opposed to distinct regions (Ruano et al., 2000; Yu et al., 2006) and this is likely affecting the comparability of results between studies. Furthermore, previous studies have been conducted on the rat and monkey but none on the mouse hippocampus. As discussed within the same section, specifically at the third paragraph of the discussion, the age-related changes of hippocampal α1 subunit expression show opposite trends for the rat and monkey (Gutiérrez et al., 1996; Yu et al., 2006). Previous studies have reported conflicting findings and species differences regarding age-related GABA signaling changes in other brain regions as well (Yu et al., 2006; Loerch et al., 2008; Rissman and Mobley, 2011; Bañuelos et al., 2014; Liguz-Lecznar et al., 2015; Pandya et al., 2018). Therefore, deviations of our findings from currently reported literature might also be because of interspecies variability. Another point to consider is that changes in mRNA do not always correspond to change in protein expression (Greenbaum et al., 2003; Liu et al., 2016). However, both the rat and monkey studies discussed above have examined the age-related alterations of α1 subunit expression at the protein level with contradictory outcomes, which is likely because of differences in experimental design, methods, and analysis.

Changes in the GABA_A_R subunit composition may result in changes of the cellular location of receptors, the type of GABAergic inhibition and pharmacokinetic properties of the receptor (Sieghart et al., 1999; Sigel and Steinmann, 2012; Sieghart and Savić, 2018). Therefore, this study provides us with important information on how GABA_A_R function is affected in the aging brain. This needs to be taken into consideration when targeting this receptor as a treatment option for age-related neurologic disorders. For example, the elderly are more sensitive to the side effects of benzodiazepines (Nikaido et al., 1990; Klein-Schwartz and Oderda, 1991), which are allosteric modulators of GABA_A_R function. Benzodiazepines are commonly used as therapeutic agents for the treatment of anxiety (Vajda and Burrows, 1983), depression (Johnson, 1985), and insomnia (Simon and VonKorff, 1997). Age-specific alterations of the GABA_A_R subunits throughout the brain have to be taken into consideration because they might influence the effect of these agents. Although in the mouse hippocampus this is not the case, as we did not observe age-related GABA_A_R subunit expression changes, there is an urgent need to examine the GABA_A_R system in the human hippocampus and other brain areas. A recent study showed that the GABA_A/B_R subunits and transporters are robust against age-related alterations in most human cortical brain regions examined except the superior temporal gyrus, suggesting a brain region-specific vulnerability of the system (Pandya et al., 2018). No age-related changes in total GABA_A_ receptor binding or agonist affinity have been reported in the rat brain, but findings related to age-dependent inhibitory activity are controversial and might show region-specific vulnerability as well (Wenk et al., 1991). Some rat studies have not demonstrated age-related changes in hippocampal inhibitory synaptic potentials (Ruano et al., 1991), whereas others suggest decreased inhibition in the hippocampus (Potier et al., 2006). Contrary to this the prefrontal cortex exhibits increased inhibition with age (Luebke et al., 2004; Schmidt et al., 2010; Lehmann et al., 2012; Cheng and Lin, 2013; Bañuelos et al., 2014). In addition, several studies reported changes in hippocampal synaptic transmission, neuronal subtype-specific cellular loss with a selective regional pattern that might lead to cognitive alterations during normal aging (Segal, 1982; Potier et al., 1992; Barnes, 1994; Vela et al., 2003; Gerrard et al., 2008; Koh et al., 2014).

Young rodents are often used as model organisms to study age-related disorders, but it is advisable to model these disorders in older mice as molecular and cellular changes occur with age. However, research with aged mice is costly, time-consuming and because aging-related health problems raise ethical concerns. Hence, observing changes in GABA_A_R subunit composition with aging will provide us with a better understanding of GABA_A_R signaling in the aged brain, and provide better characterized mouse models for future aging experiments. Studies like this are necessary to understand the importance of age in study design and interpretation of results.

In summary, our findings suggest that hippocampal GABA_A_R subunit composition is robustly preserved during aging in mice, except the localized alterations of the α2 subunit expression in the CA1 and CA3 regions. However, more studies are needed to understand the complete picture of age-related GABAergic remodeling. Age-dependent changes in GABA levels, other GABAergic signaling components and GABA_A_R subunit alterations in other brain areas might also underlie age-dependent susceptibility to, and influence disease progression in, conditions in which the fine balance of excitation and inhibition is impaired such as Alzheimer’s disease, epilepsy, or schizophrenia. With increasing life expectancy and an aging population, understanding the mechanisms and consequences of aging is critically important and could help in designing new preventive and therapeutic options for age-related disease conditions.

## Synthesis

Reviewing Editor: Lorna Role, NINDS Decisions are customarily a result of the Reviewing Editor and the peer reviewers coming together and discussing their recommendations until a consensus is reached. When revisions are invited, a fact-based synthesis statement explaining their decision and outlining what is needed to prepare a revision will be listed below. The following reviewer(s) agreed to reveal their identity: Sheryl Smith.

Both reviewers acknowledged significant improvements in the R1 manuscript and felt that most issues were addressed in a satisfactory manner. The reviewers noted that regional immunohistochemical images are now included and that the authors state that these data reveal localized changes in alpha-2 staining in CA1 hippocampus.

However, for the manuscript to be publishable, there needs to be a quantitative evaluation of the immunofluorescence data comparing across age groups. The apparent decrease in alpha-2 expression that they have noted in the Results section must be quantified. Apparently, there is decreased alpha 2 expression around the soma, proximal dendrites and axon initiating segment in ageing brains, which one would expect to have consequences for neuronal excitability, and potentially cognition, across age groups.

The authors should quantify changes (or lack thereof) and include at least a short discussion of the potential physiological consequences.

It is also misleading that the authors (still) include statements that claim that there are no age-related changes in the expression levels of alpha2 in CA1 hippocampus (abstract, lines 228-229 of page 10 of the Discussion and elsewhere) whereas in the Results section of the text they claim that this subunit is decreased in CA1 hippocampus.

The data must be quantified and the changes-- whether or not significant -- should be presented and the results discussed in the manuscript. This issue needs to be clarified and the authors should be consistent in the reporting of the data across all sections of the manuscript ( abstract, results, discussion).
